# Incidence and risk factors of post-transplant diabetes mellitus after kidney transplantation: a systematic review and meta-analysis

**DOI:** 10.3389/fendo.2026.1838424

**Published:** 2026-05-25

**Authors:** Ni Chen, Yuli Chen, Qiuhong Liu, Ying Shu

**Affiliations:** 1 Department of Nephrology, The Third People’s Hospital of Chengdu, Chengdu, Sichuan, China; 2 Health Management Center, The Third People’s Hospital of Chengdu, Chengdu, Sichuan, China; 3 Veterans’ Ward, The First Veterans Hospital Of Sichuan Province, Chengdu, Sichuan, China

**Keywords:** incidence, kidney transplantation, meta-analysis, PTDM, risk factors

## Abstract

**Objective:**

In order to investigate the incidence rate of post-transplant diabetes mellitus (PTDM) patients and identify the risk factors, we conducted a systematic review of studies published after 2014.

**Data sources:**

PubMed, EMBASE, Cochrane Library, as well as Web of Science were comprehensively retrieved until June 24, 2024.

**Selection of studies:**

Extracted data via EndNote reference management software and evaluated the risk of bias through the Newcastle-Ottawa Scale (NOS).

**Data extraction:**

Statistical analyses were enabled by STATA 15. Heterogeneity was evaluated through the Q test 、the I² statistic sensitivity and subgroup analyses.

**Data summary:**

26 studies from 15 countries involving 8, 727 participants were encompassed. The overall incidence of PTDM was 20%. Meta-analysis revealed that advanced age (>50), hepatitis C virus (HCV) infection, polycystic kidney disease, high body mass index (BMI), high fasting plasma glucose (FPG), hypertriglyceridemia, as well as hypercholesterolemia were significantly related to elevated risk of PTDM (P < 0.05). Meta-analysis of case-control studies further confirmed that advanced age (OR = 1.07, 95% CI: 1.04-1.09), high BMI (OR = 1.23, 95% CI: 1.08-1.39), and hypertriglyceridemia (OR = 1.01, 95% CI: 1.00-1.02) were significant risk factors. Cohort studies additionally identified advanced age, high BMI, high FPG, family history of diabetes, hypertriglyceridemia, and HCV infection as significant risk factors.

**Conclusions:**

The incidence of PTDM after kidney transplantation is approximately 20%. Advanced age, high BMI, family history of diabetes, high FPG, hypertriglyceridemia, and HCV infection are significant risk factors. Among these, BMI, glucose, lipid abnormalities, and HCV infection are modifiable.

**Systematic review registration:**

https://www.crd.york.ac.uk/prospero/, identifier CRD42024585070.

## 1 Introduction

Kidney transplantation is the optimal renal replacement therapy for the end-stage renal disease (ESRD) population, substantially ameliorating survival and quality of life (1, 2). With the continuous advancement of surgical techniques, the incidence of early postoperative complications has markedly decreased (1, 3). However, long-term complications, like post-transplant diabetes mellitus (PTDM), have become increasingly prominent (4, 5). Reported incidence rates of PTDM vary considerably, ranging from 2% to 50% (6–9). PTDM not only impairs allograft function and predisposes to graft dysfunction and rejection (10), but also significantly elevates the risk of cardiovascular events and infections (5). Therefore, identifying risk factors for PTDM, particularly those amenable to intervention, is of paramount importance for reducing its incidence and improving post-transplant quality of life (11).

In recent years, PTDM has attracted considerable attention in both transplant medicine and endocrinology. Numerous prospective and retrospective studies have investigated its underlying mechanisms and risk factors, highlighting the relevance of host-related characteristics like age, race, obesity, pre-transplant glucose metabolism abnormalities, and family history of diabetes (10–13). In addition, immunosuppressive regimens administered after transplantation are regarded as among the most influential clinical determinants, with calcineurin inhibitors (e.g., tacrolimus) and glucocorticoids demonstrating particularly pronounced diabetogenic effects. Although current evidence presents valuable insights into PTDM prevention and management, substantial heterogeneity exists across studies in terms of diagnostic criteria, follow-up duration, patient characteristics, and statistical methodology, resulting in considerable inconsistency among findings. For instance, while certain investigations have demonstrated a significant association between tacrolimus use and increased PTDM risk, others have failed to detect such a relationship (12, 14, 15). Furthermore, population-specific differences, like the genetic predisposition and lifestyle patterns observed among African Americans, influence both the incidence and distribution of PTDM risk factors (10, 11), thereby limiting the generalizability and clinical applicability of existing evidence.

At present, there remains a lack of comprehensive syntheses quantifying both the incidence and determinants of PTDM, particularly with respect to the magnitude and heterogeneity of associated risk factors. Several systematic reviews to date have been largely descriptive, offering limited statistical evidence to inform clinical decision-making. Moreover, with the progressive optimization of immunosuppressive regimens and improvements in patient screening strategies, more recent findings differ substantially from earlier studies, raising the concern of temporal bias. Accordingly, there is a pressing need for a methodologically rigorous systematic review and meta-analysis to provide a robust summary of the overall incidence of PTDM and to clarify its independent risk factors and their relative effects, with special emphasis on modifiable determinants.

Notably, advances in immunosuppressive protocols, greater consistency in diagnostic criteria for diabetes, and improvements in follow-up and monitoring have markedly enhanced clinical practice compared with a decade ago. Earlier studies have been subject to bias arising from outdated treatment strategies and variable diagnostic definitions. Therefore, the present study focuses on literature published after 2014 to better reflect contemporary clinical practice. This study endeavors to determine the incidence of PTDM and identify its major risk factors, including but not limited to baseline demographic characteristics, metabolic status, and immunosuppressive therapy via a systematic review and meta-analysis of global literature on kidney transplantation. By integrating current evidence, our study seeks to bridge existing knowledge gaps, provide robust support for pre-transplant risk stratification and individualized prevention strategies, and ultimately improve both long-term graft survival and overall patient outcomes.

## 2 Methods

To ensure transparency and methodological rigor, this protocol has been registered in the International Prospective Register of Systematic Reviews (PROSPERO; Registration No.: CRD42024585070) and was carried out as per the PRISMA guidelines (16).

### 2.1 Inclusion and exclusion criteria

The eligibility criteria were defined based on the Population, Exposure, Comparison, Outcome (PECO) framework for observational studies, and the PICOS strategy was applied to identify studies meeting the requirements for analysis (17). Population (P): Adult patients (≥18) who underwent kidney transplantation. Exposure (E): Any potential risk factors for PTDM as reported in the encompassed studies. These encompassed demographic characteristics (e.g., age, sex), clinical factors (e.g., pre-transplant glycemic status, body mass index (BMI), hepatitis C virus (HCV) infection status), and transplant-related factors (e.g., immunosuppressive regimens/dosages, acute rejection episodes). Comparison (C): Comparisons between exposed and non-exposed (or reference) groups as defined in the original studies, with corresponding variables for the exposure of interest. Outcome (O): Incidence of new-onset PTDM, as defined by the diagnostic criteria used in the primary studies (e.g., World Health Organization (WHO), American Diabetes Association (ADA) standards, or initiation of antidiabetic therapy). Study design (S): Cohort studies (prospective or retrospective) or case-control studies.

Exclusion criteria were: (1) studies with sample size <50 to reduce small-sample bias; (2) letters to the editor, commentaries, reviews, editorials, or expert opinions; (3) case series and case reports; (4) conference abstracts with insufficient information; (5) non-human studies; (6) unpublished or non-peer-reviewed articles.

### 2.2 Search strategy

Two investigators (N.C. and YL.C.) independently conducted systematic searches of PubMed, EMBASE, the Cochrane Database of Systematic Reviews, and Web of Science, covering all records from database inception to June 24, 2024. The search strategy encompassed the following key terms: Kidney Transplantation, Renal Transplantation, kidney graft*, kidney transplant*, Diabetes Mellitu*, Diabet*, Cohort studies, Cohort Studie*, Concurrent Stud*, Incidence Stud*, Prospective Studies, Prospective Studie*, Retrospective Studies, Retrospective Studie*, ex post facto design, post, newonset. Details are provided in the **Supplementary Table 1**.

### 2.3 Literature screening

All retrieved records were imported into EndNote. Duplicate publications were removed after automated and manual checks. Considering the substantial advancements in immunosuppressive regimens, diagnostic criteria, and follow-up strategies for kidney transplant recipients, our study only encompassed studies published from 2014 onward to ensure relevance to current clinical practice (18). Studies were first screened by title and abstract, followed by full-text review to determine final eligibility. Any discrepancies were addressed via discussion or arbitration by a third reviewer (S.Y.).

### 2.4 Data extraction

Before data extraction, a standardized electronic data collection form was developed. Extracted information encompassed: study title, first author, publication year, study design, country of corresponding author, participant age, total sample size, number of PTDM cases, diagnostic criteria for PTDM, diagnostic timeframe, data sources, and follow-up duration. The primary outcomes of interest were the incidence of new-onset PTDM following kidney transplantation, relative risks (RRs), odds ratios (ORs), as well as 95% confidence intervals (CIs).

Data were extracted independently by two investigators, with cross-checking for consistency. Any disagreements were adjudicated by a third investigator (N.C., YL.C., or S.Y.).

### 2.5 Quality assessment

The quality of encompassed studies was assessed independently by two reviewers using the Newcastle-Ottawa Scale (NOS). The NOS evaluates the representativeness of the exposed cohort, selection of the non-exposed cohort, ascertainment of exposure, demonstration that the outcome of interest was not present at study initiation, comparability of cohorts based on study design or analysis, assessment of outcome, adequacy of follow-up duration for outcomes to occur, and adequacy of cohort follow-up. Each item can score up to one point, except comparability (up to two). Studies scoring 7–8 were considered of moderate quality, whereas those scoring 9 were considered high quality. Disagreements were addressed via consultation with a third reviewer (S.Y.).

### 2.6 Statistical analysis

All statistical analyses were conducted via STATA 15. Heterogeneity was assessed via the I² statistic and Q test. A fixed-effects model was applied when heterogeneity was low (I² <50% and P >0.05); otherwise, a random-effects model was utilized. For studies with evident clinical heterogeneity, subgroup and sensitivity analyses were performed. Subgroup analyses were stratified by clinical characteristics of study populations, like age, sex, and comorbidities, to explore differences in PTDM incidence and risk factors across subgroups. Publication bias was evaluated through funnel plots and Egger’s test, with funnel plot asymmetry or p <0.05 denoting significant publication bias.

## 3 Results

### 3.1 Search results

**Figure 1:**
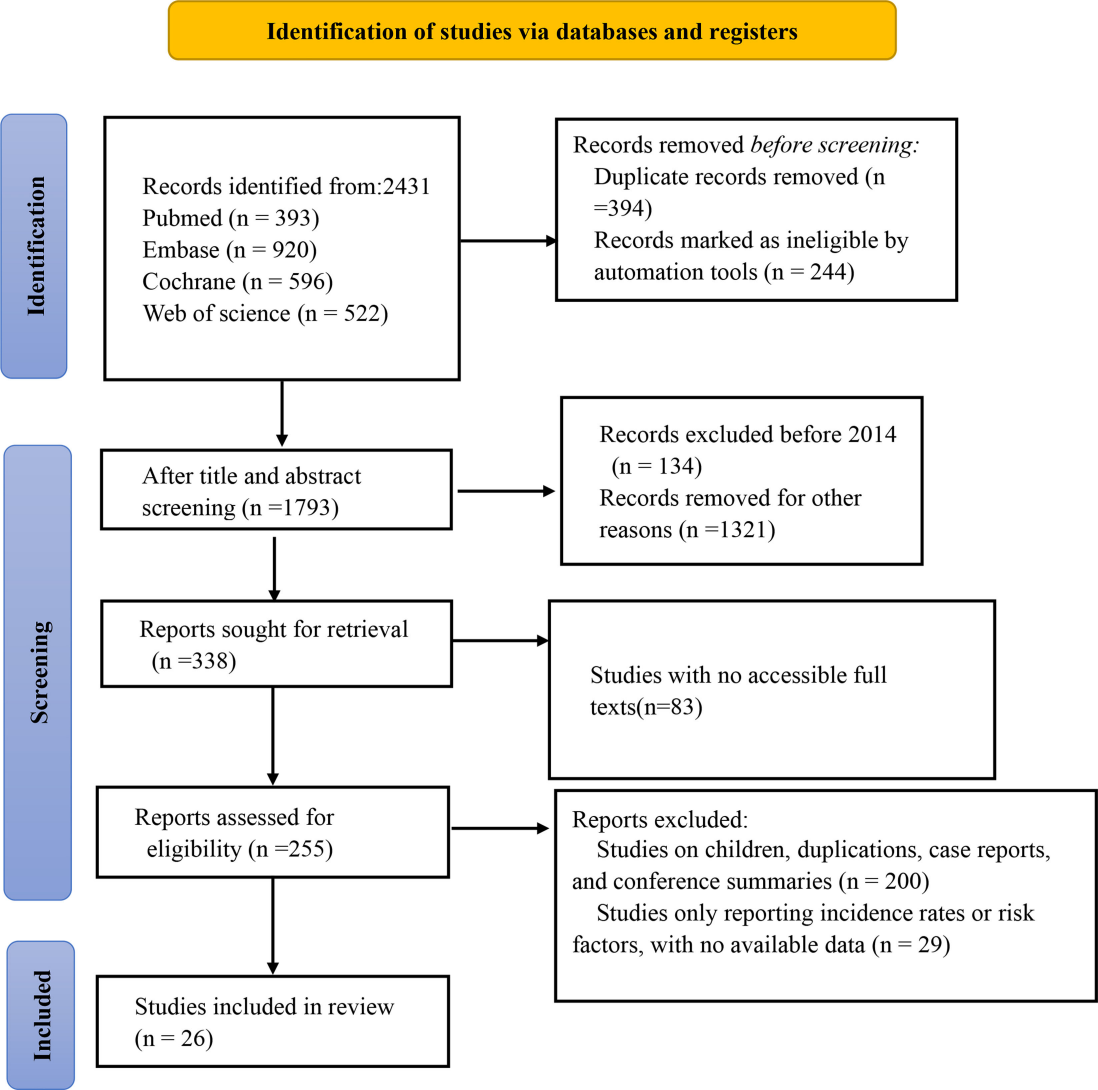
PRISMA flowchart.

A systematic search was conducted from database inception to June 24, 2024, yielding 2, 431 records: PubMed (n=393), EMBASE (n=920), Cochrane Database of Systematic Reviews (n=596), and Web of Science (n=522). After removal of duplicates (n=394) and records automatically deemed ineligible by screening tools (n=244), 1, 793 studies proceeded to title and abstract screening. At this stage, 134 studies published before 2014 were removed, and 1, 321 records were excluded for other reasons, leaving 255 studies for further assessment. Subsequently, 200 records were excluded because they focused on pediatric populations, were duplicates, case reports, or conference abstracts. 55 studies remained eligible for full-text review. Of these, 29 were removed because they reported only incidence or risk factors without extractable data. Ultimately, 26 studies were encompassed (8, 14, 15, 19–41). Details of the selection process are illustrated in **Figure 1**. PRISMA flowchart.

### 3.2 Characteristics of the encompassed studies

In total, 26 studies were encompassed (**Supplementary Table 2** (8, 14, 15, 19–41);), encompassing 8, 727 participants across 15 countries worldwide. All encompassed studies were cohort or case-control designs, with sample sizes of 77 (27) to 723 (34) participants. The mean age at PTDM diagnosis was 47.96(40.22 ± 6.34). The earliest reported time of diagnosis was 45 days post-transplant, with the longest follow-up reaching 10 years. Several studies further stratified participants by underlying primary renal disease, among which autosomal dominant polycystic kidney disease (ADPKD) was frequently reported as a cause of ESRD. Regarding immunosuppressive regimens, although some studies examined the potential effects of glucocorticoids, mTOR inhibitors, and anti-thymocyte globulin (ATG) on glucose metabolism, the majority of research primarily focused on the relation of tacrolimus to PTDM risk. Due to the lack of uniform standards for immunosuppressant use across the included studies, for example, six studies involved antimetabolites, five involved mTOR inhibitors, and four involved biologic agents, and given that some studies reported data as percentages while others used trough concentrations, only three studies provided extractable data for meta-analysis. For this reason, other immunosuppressants were not subjected to correlation analysis in the present study.

### 3.3 Quality assessment of the selected studies

The methodological quality of the 26 encompassed cohort studies was rated via the NOS (**Supplementary Table 3**). Overall, studies scored lowest in the “outcome assessment” domain, particularly due to inadequate follow-up (e.g., insufficient duration or high loss to follow-up).

Specifically, 11 studies scored 7–8 points, indicating moderate quality. Studies with a score of 7 were mainly downgraded for insufficient follow-up or unclear definition/measurement of outcomes (14, 29). Studies scoring 8 points were typically limited by inadequate control for confounding at baseline, incomplete follow-up (high attrition rates), or lack of blinding in outcome assessment (15, 20, 23, 25, 28, 30, 31, 33, 36). The remaining 15 studies (57.7%) achieved a NOS score of 9, reflecting high methodological quality.

### 3.4 Forest plot of incidence

**Figure 2:**
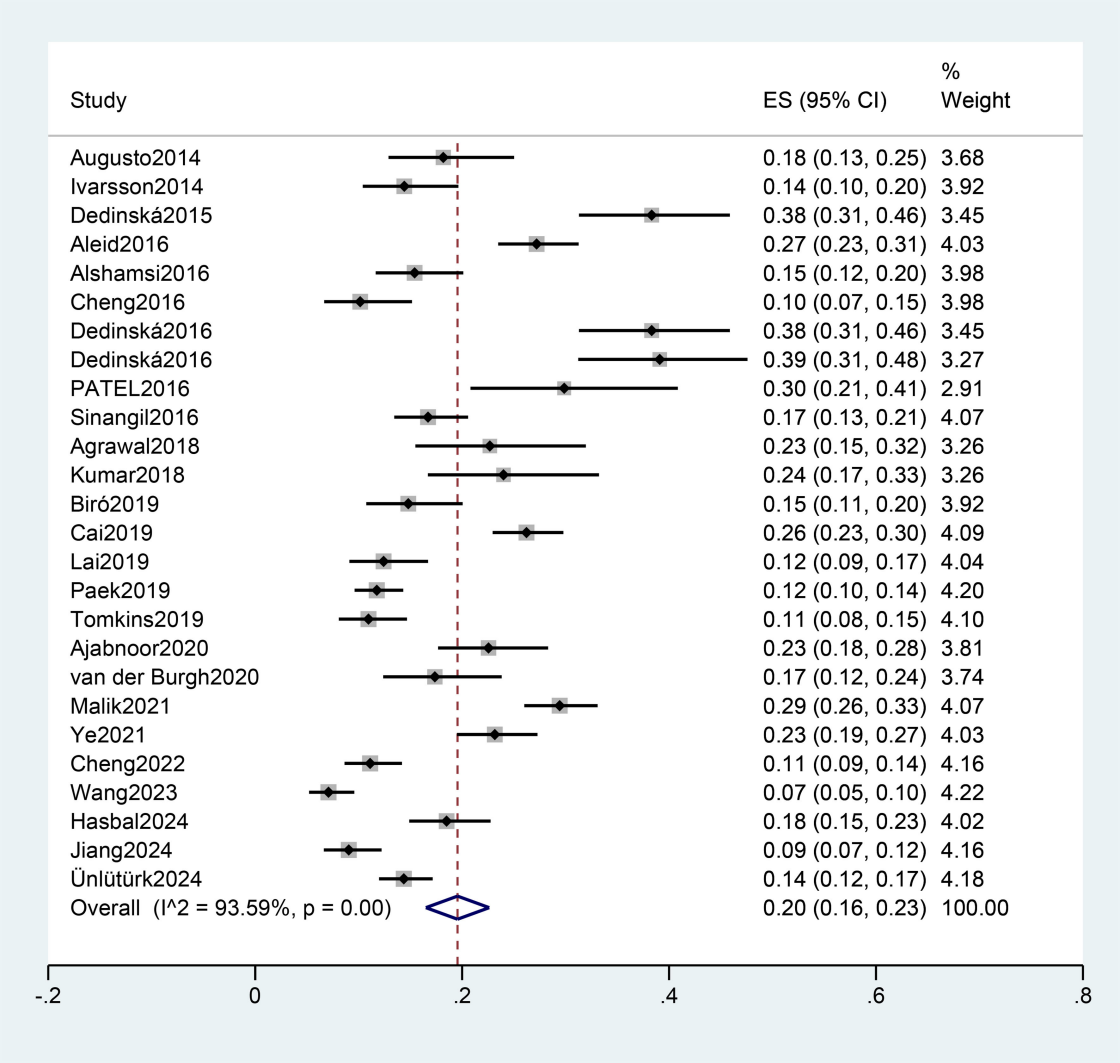
Forest plot of PTDM incidence.

Our meta-analysis of the 26 encompassed studies demonstrated an overall PTDM incidence of 20% (95% CI: 0.16-0.23). Substantial heterogeneity was observed (I²=93.59%, P < 0.05), prompting further subgroup and sensitivity analyses (**Figure 2**).

### 3.5 Subgroup analysis

A subgroup analysis was conducted across 26 studies. The heterogeneity tests for all subgroups revealed I² > 50% and P < 0.05, indicating that the examined variables were not the principal sources of overall heterogeneity. Nevertheless, variations in incidence were observed among certain variables, which provide informative insights.

In the age-stratified analysis, the incidence of PTDM was higher in participants aged >50 (ES = 0.22, 95% CI: 0.17-0.28) in comparison to those aged ≤50 (0.17, 95% CI: 0.13-0.21). With respect to sex, the incidence rates were comparable between males (0.19, 95% CI: 0.16-0.22) and females (0.18, 95% CI: 0.15-0.22). Among participants with comorbid HCV infection or ADPKD, the incidence of PTDM was 0.29 (95% CI: 0.19-0.40 and 0.20-0.38, respectively), both markedly higher than that in those without such comorbidities (0.20 and 0.20, respectively). Regarding BMI, the highest incidence was observed in individuals with BMI ≥28 kg/m² (ES = 0.25, 95% CI: 0.15-0.34), whereas those with BMI ≤23.9 kg/m² exhibited the lowest incidence (0.13, 95% CI: 0.09-0.16), suggesting a trend of increasing PTDM risk with rising BMI. In participants with fasting plasma glucose (FPG) >5.5 mmol/L, the incidence was 0.17 (95% CI: 0.10-0.23), higher than that in the ≤5.5 mmol/L group (0.14, 95% CI: 0.10-0.19). As for dyslipidemia, the incidence of PTDM was 0.22 (95% CI: 0.15-0.30) in the TG >1.7 mmol/L group and 0.23 (95% CI: 0.16-0.31) in the TC >5 mmol/L group, both exceeding the rates observed in their corresponding lower-level groups (0.20 and 0.17, respectively). Therefore, elevated FPG and hyperlipidemia are related to an increased PTDM risk. According to the timing of PTDM onset, the incidence within 3 months was 0.08 (95% CI: 0.06-0.10), within 6 months was 0.10 (95% CI: 0.08-0.12), and within 12 months was 0.12 (95% CI: 0.10-0.15) (**Table 1**).

**Table 1:** Subgroup analyses of incidence.

Subgroup analyses	Study quantity	Heterogeneity		*ES*(95%Cl)
		*I^2^*(%)	*P* value	
Age				
≤50	14	91.79	0	0.17(0.13, 0.21)
>50	12	94.32	0	0.22(0.17, 0.28)
Sex				
Male	11	89.04	0	0.19(0.16, 0.22)
Female	11	88.43	0	0.18(0.15, 0.22)
HCV				
positive	11	73.28	0	0.29(0.19, 0.40)
negatives	11	95.35	0	0.20 (0.15, 0.25)
ADPKD				
positive	11	64.98	0	0.29(0.20, 0.38)
negatives	11	93.84	0	0.20(0.15, 0.26)
BMI				
≤23.9kg/m^2^	8	88.38	0	0.13(0.09, 0.16)
24-27.9 kg/m^2^	10	85.77	0	0.20(0.17, 0.24)
≥28 kg/m^2^	4	93.24	0	0.25(0.15, 0.34)
FPG				
≤5.5mmol/l	7	91.67	0	0.14(0.10, 0.19)
>5.5mmol/l	5	92.68	0	0.17(0.10, 0.23)
TG				
≤1.7mmol/l	6	90.56	0	0.20(0.14, 0.27)
>1.7mmol/l	7	94.11	0	0.22(0.15, 0.30)
TC				
≤5mmol/l	7	94.69	0	0.17(0.11, 0.23)
>5mmol/l	5	86.50	0	0.23(0.16, 0.31)
Time				
Incidence within 3 months	4	70.90	0.02	0.08(0.06, 0.10)
Incidence within 6 months	4	5.80	0.36	0.10(0.08, 0.12)
Incidence within one year	4	73.60	0.01	0.12(0.10, 0.15)

### 3.6 Sensitivity analysis

**Figure 3:**
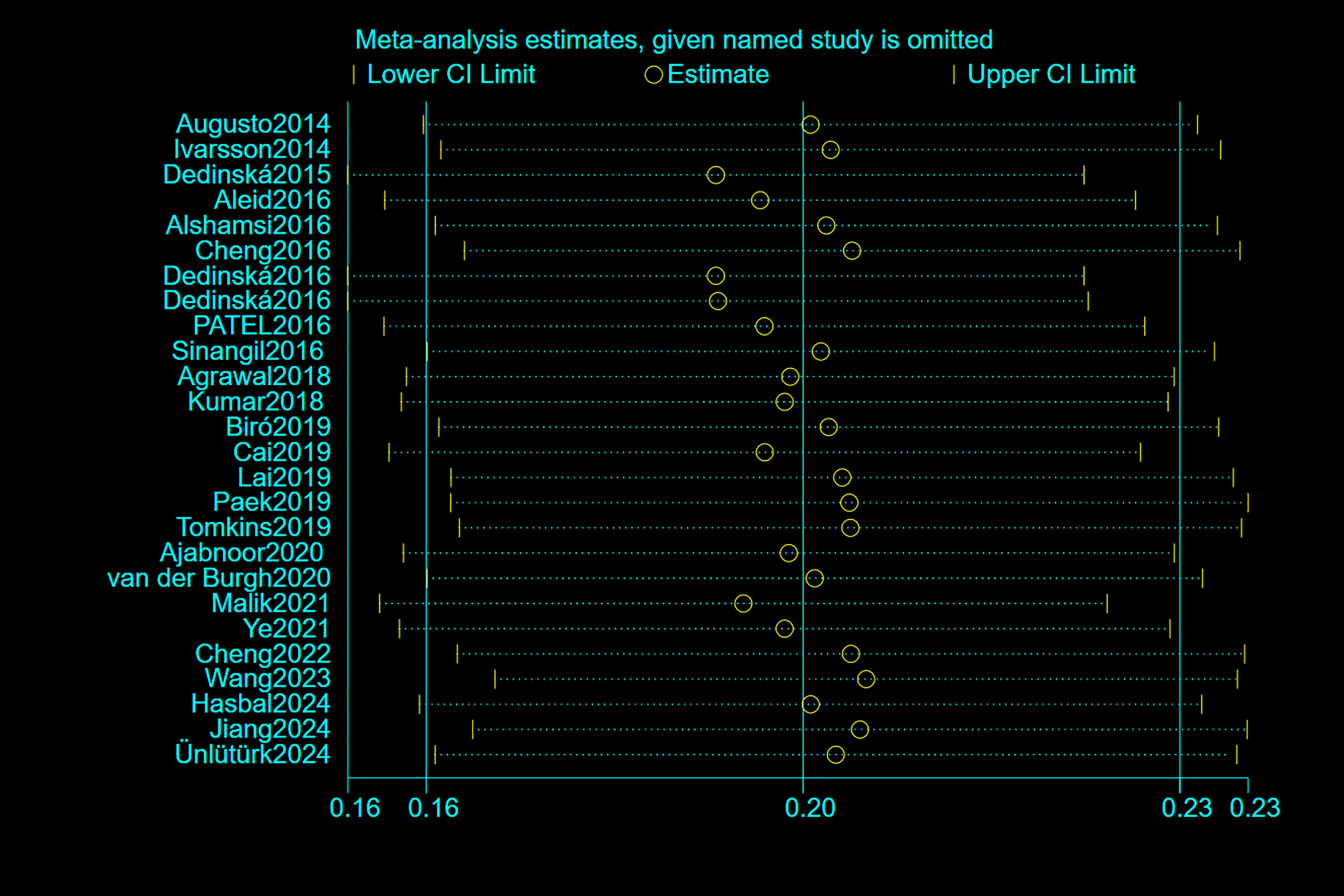
Sensitivity analysis of PTDM incidence.

In addition, sensitivity analyses were conducted. The results demonstrated that no single study exerted a significant influence on the overall outcomes of PTDM, as illustrated in **Figure 3**.

### 3.7 Publication bias

**Figure 4:**
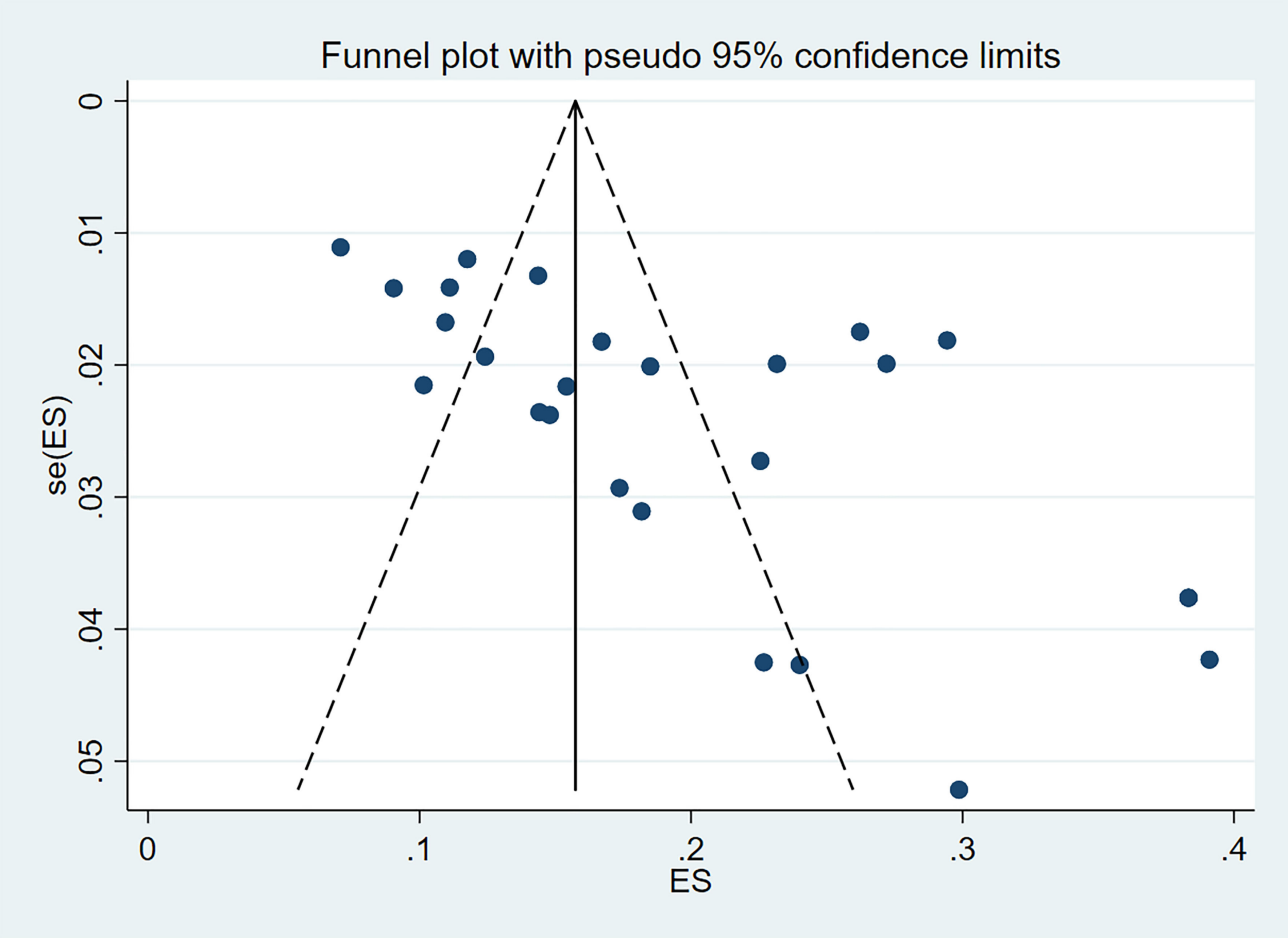
Funnel plot of PTDM incidence.

**Figure 5:**
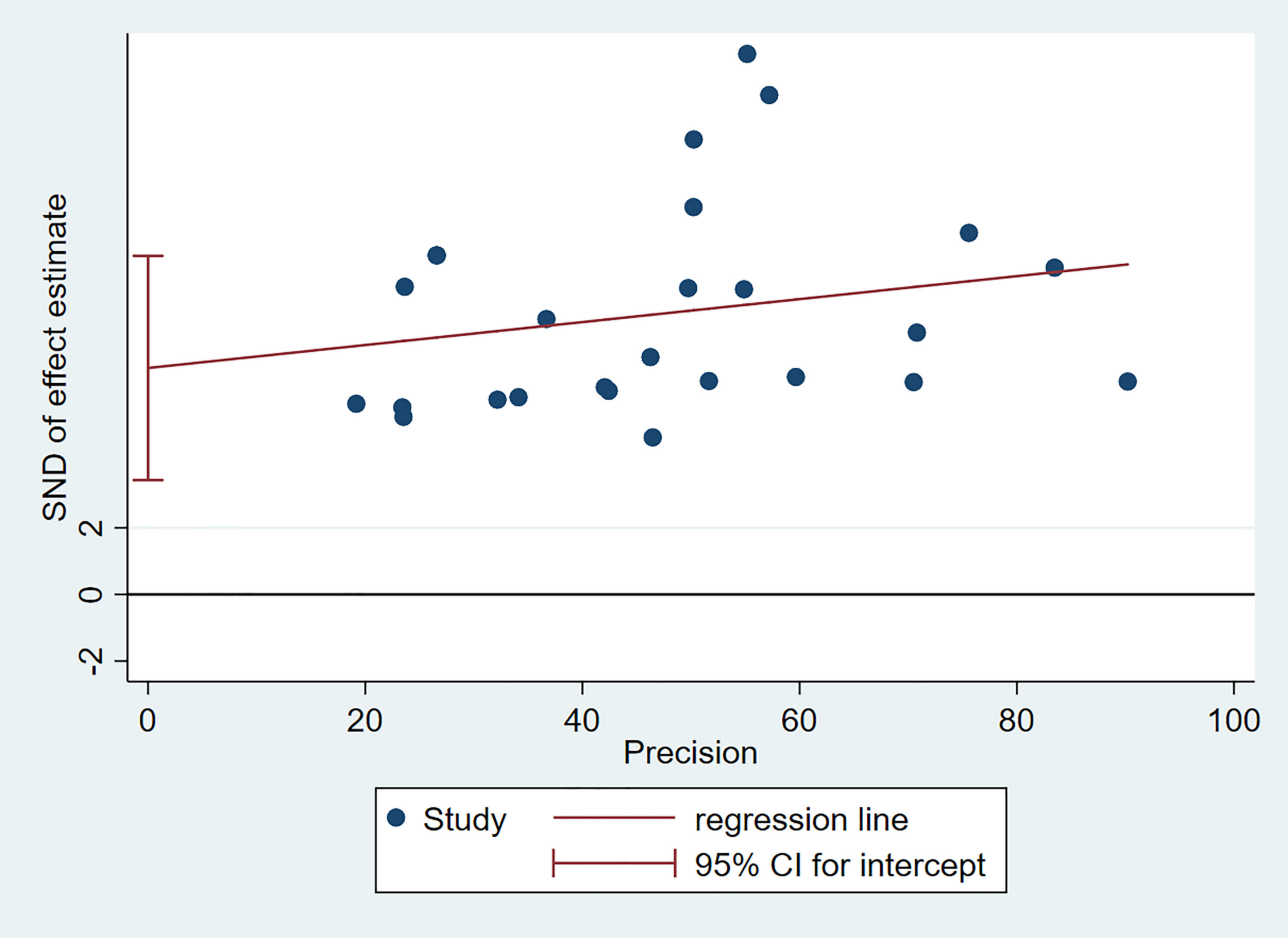
Egger’s test of PTDM incidence.

Publication bias was detected via funnel plots and Egger’s test. The funnel plot revealed an approximately symmetrical distribution of scatter points (**Figure 4**), suggesting a relatively low risk of potential publication bias. Consistently, Egger’s test indicated no publication bias, with P > 0.05 (**Figure 5**).

### 3.8 Risk factors

With respect to age, 21 studies demonstrated that older individuals (>50) had a significantly elevated PTDM risk (OR = 1.07, 95% CI: 1.04-1.09; RR = 1.09, 95% CI: 1.03-1.15). Regarding sex, five cohort studies suggested no significant difference in PTDM risk between males and females (RR = 1.39, 95% CI: 0.84-2.30). In terms of BMI, 16 studies revealed that higher BMI was related to a significantly increased PTDM risk (OR = 1.23, 95% CI: 1.08-1.39; RR = 1.14, 95% CI: 1.05-1.24).

For FPG, eight cohort studies showed that individuals with elevated FPG had a significantly higher PTDM risk (RR = 1.47, 95% CI: 1.15-1.86), whereas case-control studies did not reach statistical significance (OR = 1.06, 95% CI: 0.99-1.13). Cohort studies indicated that a family history of diabetes was significantly related to increased PTDM risk (RR = 4.05, 95% CI: 1.15-14.21), whereas case-control studies failed to demonstrate significance (OR = 2.04, 95% CI: 0.86-4.83).

As for triglycerides (TG), eight studies revealed that individuals with elevated TG levels had an increased PTDM risk (OR = 1.01, 95% CI: 1.00-1.02; RR = 1.32, 95% CI: 1.06-1.63). Patients with HCV infection exhibited a significantly elevated PTDM risk (RR = 1.77, 95% CI: 1.30-2.41). For tacrolimus blood concentration, six studies reported ORs that did not reach statistical significance (OR = 1.05, 95% CI: 0.97-1.12), and a single cohort study likewise showed no significant association in RR analysis (RR = 1.45, 95% CI: 0.97-2.17).

In summary, advanced age, elevated BMI, increased FPG, family history of diabetes, higher TG levels, and HCV infection were all significantly related to an increased PTDM risk, whereas the effects of sex and tacrolimus concentration remain inconclusive(**Table 2**). It should be specifically noted that among the six studies addressing tacrolimus concentration, only one used the area under the curve (AUC) for whole-blood tacrolimus (37), while the remaining studies used tacrolimus trough concentrations.

**Table 2:** Subgroup analyses of risk factors.

Subgroup analyses	Study quantity	OR/RR	Heterogeneity		RR (95%Cl)	EC
			*I^2^* (%)	*P* value		
Age						
	12	OR	64.4	0.001	1.07 (1.04, 1.09)	Older/younger
	9	RR	91.0	0.000	1.09 (1.03, 1.15)	Older/younger
Sex						
Male: Female	5	RR	83.9	0.000	1.39 (0.84, 2.30)	
BMI						
	11	OR	83.1	0.000	1.23 (1.08, 1.39)	High/Low
	5	RR	69.3	0.011	1.14 (1.05, 1.24)	High/Low
FPG						
	4	OR	89.3	0.000	1.06 (0.99, 1.13)	High/Low
	4	RR	80.8	0.001	1.47 (1.15, 1.86)	High/Low
Family history of diabetes						
	3	OR	68.3	0.043	2.04 (0.86, 4.83)	Yes/No
	4	RR	89.7	0.000	4.05 (1.15, 14.21)	Yes/No
TG						
	4	OR	75.9	0.006	1.01 (1.00, 1.02)	High/Low
	4	RR	57.2	0.072	1.32 (1.06, 1.63)	High/Low
HCV						
	5	RR	60.0	0.040	1.77 (1.30, 2.41)	Yes/No
Tac						
	5	OR	83.3	0.000	1.05 (0.97, 1, 12)	High/Low
	1	RR	NA		1.45 (0.97, 2.17)	High/Low

## 4 Discussion

This study systematically integrated 26 multicenter cohort studies involving 8, 727 kidney transplant recipients to comprehensively evaluate the incidence and associated risk factors of PTDM, thereby providing authoritative evidence-based insights. The overall incidence of PTDM reached 20%. Subgroup analyses further demonstrated that advanced age, elevated BMI, increased FPG, higher TG levels, HCV infection, and ADPKD were all significantly related to an increased PTDM risk, underscoring PTDM as a postoperative metabolic complication of considerable clinical importance that warrants heightened awareness.

The findings revealed that the overall incidence of PTDM was notably high, with patients aged >50 years exhibiting a markedly greater incidence compared with younger individuals, highlighting age as one of the most critical non-modifiable risk factors. The substantial metabolic burden imposed by transplantation and immunosuppressive therapy in elderly patients (42–44)places them at heightened risk for PTDM. BMI, as a modifiable factor, was positively correlated with PTDM, further supporting the role of obesity and metabolic syndrome in driving postoperative disturbances in glucose metabolism (45–50). Adipose tissue produces adipokines, including leptin, tumor necrosis factor-α (TNF-α), interleukins (ILs), and adiponectin (51). Among these mechanisms, chronic inflammation plays a central role. Pro-inflammatory cytokines such as TNF-α and IL-6, the latter being associated with impaired glucose tolerance and demonstrated to be a predictor of Type 2 diabetes mellitus (T2DM) (52), are particularly important. In addition, obesity-induced chronic inflammation directly impairs pancreatic β-cell function and reduces glucose uptake and utilization in hepatic and skeletal muscle tissues (49, 50). These mechanisms constitute the pathological basis underlying the high incidence of PTDM in individuals with obesity.

Moreover, the observed associations between dyslipidemia (particularly hypertriglyceridemia and hypercholesterolemia) and PTDM suggest that lipid metabolic dysfunction possibly contributes to PTDM through mechanisms of metabolic imbalance and insulin resistance (53). Elevated preoperative FPG was also predictive of higher PTDM risk, indicating that impaired glucose metabolism before transplantation is an important prognostic marker (54). Elevated FPG before transplantation indicates that recipients may already exhibit insulin resistance and/or insufficient β-cell functional reserve (55). When such individuals with underlying metabolic defects are subsequently exposed to the strong diabetogenic pressures after transplantation, such as glucocorticoid-induced exacerbation of insulin resistance and tacrolimus-induced impairment of β-cell function, their compensatory mechanisms are readily overwhelmed, resulting in persistent hyperglycemia and ultimately progression to PTDM (55, 56). Accordingly, optimization of glycemic control before surgery lowers the PTDM risk (57).

HCV infection significantly increased the PTDM risk, which aligns with established mechanisms involving insulin resistance and β-cell injury (58, 59). Hepatitis viruses, through pro-inflammatory cytokine release and hepatic metabolic disturbances, can induce systemic metabolic imbalance (60), with the risk further amplified under the influence of immunosuppressive therapy. These findings underscore the importance of pre-transplant screening and active management of HCV infection as a critical preventive strategy (27).

Patients with ADPKD were also found to have a higher incidence of PTDM, potentially attributable to their unique renal pathology and associated metabolic abnormalities (61). Nevertheless, the underlying mechanisms remain insufficiently elucidated, warranting further mechanistic research.

Tacrolimus, as a cornerstone immunosuppressant, has been widely implicated in PTDM pathogenesis (44, 62). The principal mechanism underlying tacrolimus-induced PTDM is the direct impairment of pancreatic β-cell function. Tacrolimus binds to and inhibits calcineurin within β cells, thereby critically blocking the calcineurin/nuclear factor of activated T cells (NFAT) signaling pathway (63). This inhibition not only directly downregulates insulin gene expression but also disrupts mitochondrial function and suppresses key transcriptional coactivators, such as cAMP response element-binding protein (CREB), ultimately leading to a marked reduction in insulin biosynthesis and secretion (63, 64). Secondary mechanisms include tacrolimus-mediated inhibition of glucose uptake in skeletal muscle and adipose tissue, thereby exacerbating peripheral insulin resistance (63). Through interference with these signaling pathways related to insulin resistance and β-cell function, immunosuppressive agents collectively contribute to the development of PTDM (65). Prolonged exposure to high doses of tacrolimus, particularly with a high cumulative dose, exerts adverse effects on insulin secretion and glucose metabolism. High-dose and long-term use of tacrolimus impairs pancreatic β-cell function, thereby increasing the risk of diabetes mellitus. A study by Ajabnoor et al. confirmed that a trough concentration>10 ng/mL during the first three months significantly increases the incidence of PTDM (36)Nevertheless, this meta-analysis found no significant relation of tacrolimus blood concentration to PTDM risk. Our findings are inconsistent with some previous studies. This discrepancy may be attributable to the limited amount of included data, as only six studies were eligible for meta-analysis; the scarcity of original literature may have introduced statistical error. In addition, prevalence-incidence bias may have influenced the analysis of incident cases, suggesting that PTDM development is not solely dependent on drug exposure levels but rather influenced by multiple factors, including genetic background, pharmacogenomic variability, combination immunosuppressive regimens, and baseline metabolic status. This emphasizes the need for cautious interpretation of single-parameter associations with PTDM risk and highlights the importance of future high-quality prospective studies incorporating pharmacogenomics to validate these findings.

Several previous systematic reviews and meta-analyses have investigated the risk factors for PTDM. For example, Xia et al. (2020) reported an overall incidence of approximately 18% (6), whereas the most recent meta-analysis by Du et al. (2024) indicated an incidence of 21% (54). The present study yielded incidence rates broadly consistent with prior findings but, by including only studies published after 2014, ensured alignment with contemporary immunosuppressive regimens and diagnostic criteria, thereby enhancing clinical relevance. Furthermore, this study provided quantitative risk estimates for additional factors like family history of diabetes, HCV infection, and ADPKD, offering a more comprehensive delineation of the risk factor spectrum. From a temporal perspective, the incidence is relatively higher within the first 3 months, and the cumulative incidence increases over time. Thus, this study not only updates the evidence base on PTDM incidence but also identifies several modifiable risk factors, providing more targeted guidance for clinical risk stratification and preventive strategies.

High heterogeneity (I² > 90%) was observed in this meta-analysis, largely attributable to variations across encompassed studies in baseline patient characteristics, immunosuppressive protocols, diagnostic criteria for PTDM, follow-up duration, and geographic distribution. Although subgroup analyses did not fully account for the observed heterogeneity, they nonetheless highlighted the predictive value of several risk factors for PTDM incidence. This underscores the necessity of individualized risk assessment and management in clinical practice.

Glucocorticoids are also one of the potential contributors to PTDM. It is well established that glucocorticoids may induce drug-related diabetes (66). However, due to heterogeneity in the original studies, particularly differences in treatment regimens (e.g., maintenance therapy versus pulse therapy), a correlation analysis cannot the performed in this study, which may have contributed to inconsistencies in the statistical results. The meta-analysis of tacrolimus was limited to trough concentration data from only six studies, with considerable variation in measurement timing, ranging from single time-point assessments to four-week averages or AUC. Notably, none of the included studies provided extractable data on cumulative dose or long-term exposure duration, both of which have been established as key determinants of PTDM risk. Therefore, the findings regarding tacrolimus should be interpreted with caution, and future prospective studies incorporating pharmacokinetic exposure metrics (e.g., AUC and cumulative dose) are warranted to validate these associations.

It is also noteworthy that most included studies were observational cohorts, which may introduce selection bias and confounding. Differences between OR and RR estimates for certain risk factors further caution against inferring causality. Variability in follow-up duration and relatively high attrition rates may also affect the precision and stability of results. Additionally, the deliberate restriction to studies published after 2014 enhances the applicability of findings to contemporary practice but may limit the availability of long-term follow-up data.

## 5 Conclusion

Kidney transplantation remains the most effective treatment for ESRD. Nevertheless, postoperative complications continue to pose major challenges to long-term graft survival and patient prognosis. This systematic review and meta-analysis demonstrated that the overall incidence of PTDM is approximately 20%, with risk factors including advanced age, elevated BMI, family history of diabetes, increased FPG, hypertriglyceridemia, and HCV infection. Notably, BMI, dysglycemia, dyslipidemia, and HCV infection represent modifiable risk factors.

Therefore, comprehensive preoperative risk assessment is imperative. Regular monitoring of fasting blood glucose is recommended. For patients with a family history of diabetes, more proactive management should be implemented; in addition to achieving target fasting glucose levels, controlling postprandial glucose may yield further benefits. Blood lipids should be actively maintained within the normal range. Weight management should be maintained throughout the treatment course, as appropriate weight control may help reduce the risk of PTDM. Some studies suggest that reducing waist circumference may also help lower PTDM risk, although further research is needed. In addition, early screening for HCV is crucial, and infected individuals should receive prompt treatment to reduce the risk of PTDM, alongside individualized immunosuppressive regimens to further mitigate this risk. Future multicenter prospective studies, particularly those integrating pharmacogenomic and metabolic profiling, are warranted to further elucidate the mechanisms underlying PTDM and to provide evidence-based strategies for precision prevention and intervention.

## Data Availability

The original contributions presented in the study are included in the article/**Supplementary Material**. Further inquiries can be directed to the corresponding author.

## References

[1] Khadjibaev F Sultanov P Ergashev D Sadikov R Djuraev J Iskhakov N . Frequency of complications after kidney transplant in the early postoperative period. Exp Clin Transplant. (2024) 22:195–9. doi: 10.6002/ect.MESOT2023.P25 38385397

[2] Strohmaier S Wallisch C Kammer M Geroldinger A Heinze G Oberbauer R . Survival benefit of first single-organ deceased donor kidney transplantation compared with long-term dialysis across ages in transplant-eligible patients with kidney failure. JAMA Netw Open. (2022) 5:e2234971. doi: 10.1001/jamanetworkopen.2022.34971. PMID: 36205998 PMC9547326

[3] Cassini MF . Surgical complications of renal transplantation. In: Understanding the complexities of kidney transplantation. London, UK: InTech (2011). p. 527–47.

[4] Borda B Lengyel C Varkonyi T Ivanyi B Keresztes C Lazar G . Early histopathological changes in new onset diabetes after kidney transplantation. Nephrol Dialysis Transplant. (2014) 29:iii203. doi: 10.1016/j.transproceed.2014.05.057. PMID: 25131129

[5] Abbas MH Ismail MI El Deeb SA Nagib AM Hassan NM Refaie AF . Effect of pretransplant hepatitis C virus on the development of new-onset diabetes mellitus after transplant in Egyptian living-donor renal allotransplant recipients at mansoura urology and nephrology center. Exp Clin Transplant. (2015) 13:6–18. doi: 10.6002/ect.2014.0090 25654411

[6] Xia M Yang H Tong X Xie H Cui F Shuang W . Risk factors for new‐onset diabetes mellitus after kidney transplantation: A systematic review and meta‐analysis. J Diabetes Invest. (2020) 12:109–22. doi: 10.1111/jdi.13317. PMID: 32506801 PMC7779280

[7] Larsen J Boerner B Shivaswamy V . Post-transplant diabetes mellitus: Causes, treatment, and impact on outcomes. Endocr Rev. (2016) 37:37–61. doi: 10.1210/er.2015-1084. PMID: 26650437 PMC4740345

[8] Malik RF Jia Y Mansour SG Reese PP Hall IE Alasfar S . Post-transplant diabetes mellitus in kidney transplant recipients: A multicenter study. Kidney360. (2021) 2:1296–307. doi: 10.34067/kid.0000862021. PMID: 35369651 PMC8676388

[9] Rossi MR Mazzali M de Sousa MV . Post-transplant diabetes mellitus: Risk factors and outcomes in a 5-year follow-up. Front Clin Diabetes Healthcare. (2024) 5:1336896. doi: 10.3389/fcdhc.2024.1336896. PMID: 38352660 PMC10863447

[10] Abdulrahman MM Idris MA Elhakimi WF Akhtar M Hammam M Aldajani AA . New-onset diabetes after transplantation among renal transplant recipients at a new transplant center; King Fahad Specialist Hospital-Dammam, Saudi Arabia. Saudi J Kidney Dis Transplantation: Off Publ Saudi Center For Organ Transplantation Saudi Arabia. (2018) 29:863–71. doi: 10.4103/1319-2442.239641. PMID: 30152423

[11] Ahmed SH Biddle K Augustine T Azmi S . Post-transplantation diabetes mellitus. Diabetes Ther. (2020) 11:779–801. doi: 10.1007/s13300-020-00790-5. PMID: 32095994 PMC7136383

[12] Ouni A Sahtout W Hadj Brahim M Azzabi A Aicha NB Mrabet S . New-onset diabetes as a complication after kidney:transplant_ incidence and outcomes.pdf>. Exp Clin Transpl. (2022) 20:129–31. 10.6002/ect.MESOT2021.P56 35384822

[13] Kirnap NG Bozkus Y Haberal M . Analysis of risk factors for posttransplant diabetes mellitus after kidney transplantation: Single-center experience. Exp Clin Transplant. (2020) 18:36–40. doi: 10.6002/ect.tond-tdtd2019.o8. PMID: 32008491

[14] Ünlütürk U Yıldırım T Savaş M Oğuz SH Fırlatan B Yüce D . Effect of post-transplant diabetes mellitus on cardiovascular events and mortality: a single‐center retrospective cohort study. Endocrine. (2024) 85:695–703. doi: 10.1007/s12020-024-03770-y 38491339

[15] Jiang Z Xu C Hou H Yang X Qian M Zuo L . Does antibiotic use increase the risk of post-transplantation diabetes mellitus? A retrospective study of renal transplant patients. Ann Transplant. (2024) 29:e943282. doi: 10.12659/aot.943282. PMID: 38685698 PMC11069324

[16] Page MJ McKenzie JE Bossuyt PM Boutron I Hoffmann TC Mulrow CD . The PRISMA 2020 statement: an updated guideline for reporting systematic reviews. BMJ. (2021). doi: 10.1136/bmj.n71. PMID: 33782057 PMC8005924

[17] Eriksen MB Frandsen TF . The impact of patient, intervention, comparison, outcome (PICO) as a search strategy tool on literature search quality: a systematic review. J Med Library Assoc. (2018) 106:420–31. doi: 10.5195/jmla.2018.345. PMID: 30271283 PMC6148624

[18] Sharif A Hecking M de Vries APJ Porrini E Hornum M Rasoul-Rockenschaub S . Proceedings from an international consensus meeting on posttransplantation diabetes mellitus: Recommendations and future directions. Am J Transplant. (2014) 14:1992–2000. doi: 10.1111/ajt.12850. PMID: 25307034 PMC4374739

[19] Augusto JF Subra JF Duveau A Rakotonjanahary J Dussaussoy C Picquet J . Relation between pretransplant magnesemia and the risk of new onset diabetes after transplantation within the first year of kidney transplantation. Transplantation. (2014) 97:1155–60. doi: 10.1097/01.tp.0000440950.22133.a1. PMID: 24686469

[20] Ivarsson KM Clyne N Almquist M Akaberi S . Hyperparathyroidism and new onset diabetes after renal transplantation. Transplant Proc. (2014) 46:145–50. doi: 10.1016/j.transproceed.2013.07.076. PMID: 24507041

[21] Dedinská I Laca Ľ Miklušica J Rosenberger J Žilinská Z Galajda P . Waist circumference as an independent risk factor for NODAT. Ann Transplant. (2015) 20:154–9. doi: 10.12659/AOT.892067 25791039

[22] Aleid H Alhuraiji A Alqaraawi A Abdulbaki A Altalhi M Shoukri M . New-onset diabetes after kidney transplantation: Incidence, risk factors, and outcomes. Saudi J Kidney Dis Transplantation: Off Publ Saudi Center For Organ Transplantation Saudi Arabia. (2016) 27:1155–61. doi: 10.4103/1319-2442.194603. PMID: 27900960

[23] Alshamsi S Basri N Flaiw A Ghamdi G Hejaili F Shaheen FA . Predictability and risk factors for development of new-onset type 2 diabetes mellitus after transplant in the Saudi population. Exp Clin Transplant. (2016) 14:271–5. doi: 10.6002/ect.2015.0230 27221718

[24] Cheng Y Li H Yang L Meng Y Liu Q Li X . The incidence and risk factors for new-onset diabetes after transplantation in kidney allograft recipients. Int J Clin Exp Med (2016) 9:22250–8.

[25] Dedinská I Laca Ľ Miklušica J Kantárová D Galajda P Mokáň M . Correlation between CMV infection and post-transplantation new-onset diabetes mellitus. Int J Organ Transplant Med. (2016) 7:173–82. PMC5054141 27721964

[26] Dedinská I Baltesová T Beňa Ĺ Čellár M Galajda P Chrastina M . Incidence of diabetes mellitus after kidney transplantation in Slovakia: Multicentric, prospective analysis. Transplant Proc. (2016) 48:3292–8. doi: 10.1016/j.transproceed.2016.09.041 27931571

[27] Patel S Gohel K Patel B . Incidences of-and risk factor for new onset diabetes after transplantation in live donor kidney transplantation: A prospective single centre study. Int J Pharm Pharm Sci. (2016) 8.

[28] Sinangil A Celik V Barlas S Sakaci T Koc Y Basturk T . New-onset diabetes after kidney transplantation and pretransplant hypomagnesemia. Prog Transplant (Aliso Viejo Calif). (2016) 26:55–61. doi: 10.1177/1526924816633949. PMID: 27136250

[29] Agrawal RK Agrawal R Agrawal P Baral A Hada R . Incidence and associated risk factors of new-onset diabetes mellitus after transplantation in renal transplant recipients: A retrospective single-center study in Nepal. Indian J Transplant. (2018) 12:103–9. doi: 10.4103/ijot.ijot_2_18. PMID: 42110536

[30] Kumar S Sanyal D Das P Bhattacharjee K Rungta R . An observational prospective study to evaluate the preoperative risk factors of new-onset diabetes mellitus after renal transplantation in a tertiary care centre in Eastern India. Indian J Endocrinol Metab. (2018) 22:610–5. doi: 10.4103/ijem.ijem_121_18. PMID: 30294568 PMC6166566

[31] Biró B Szabó RP Illésy L Balázsfalvi N Szőllősi GJ Baráth S . Regulatory T cells in the context of new-onset diabetes after renal transplant: A single-center experience. Transplant Proc. (2019) 51:1234–8. doi: 10.1016/j.transproceed.2019.03.007 31101204

[32] Cai R Wu M Xing Y . Pretransplant metabolic syndrome and its components predict post-transplantation diabetes mellitus in Chinese patients receiving a first renal transplant. Ther Clin Risk Manag. (2019) 15:497–503. doi: 10.2147/tcrm.s190185. PMID: 30936711 PMC6422405

[33] Lai X Zhang L Fang J Li G Xu L Ma J . OGTT 2-hour serum C-peptide index as a predictor of post-transplant diabetes mellitus in kidney transplant recipients. Ann Trans Med. (2019) 7:1–8. doi: 10.21037/atm.2019.10.14. PMID: 31807520 PMC6861761

[34] Paek JH Kang SS Park WY Jin K Park SB Han S . Incidence of post-transplantation diabetes mellitus within 1 year after kidney transplantation and related factors in Korean cohort study. Transplant Proc. (2019) 51:2714–7. doi: 10.1016/j.transproceed.2019.02.054. PMID: 31477423

[35] Tomkins M Tudor RM Cronin K O'Kelly P Williams Y Little D . Risk factors and long-term consequences of new-onset diabetes after renal transplantation. Ir J Med Sci. (2020) 189:497–503. doi: 10.1007/s11845-019-02112-6. PMID: 31631244

[36] Ajabnoor A Nasser M Khan N Habhab W . Evaluation of tacrolimus trough level in patients who developed post-transplant diabetes mellitus after kidney transplantation: A retrospective single-center study in Saudi Arabia. Transplant Proc. (2020) 52:3160–7. doi: 10.1016/j.transproceed.2020.05.014. PMID: 32636070

[37] van der Burgh AC Moes A Kieboom BCT van Gelder T Zietse R van Schaik RHN . Serum magnesium, hepatocyte nuclear factor 1β genotype and post-transplant diabetes mellitus: a prospective study. Nephrol Dial Transplant. (2020) 35:176–83. doi: 10.1093/ndt/gfz145. PMID: 31361318

[38] Ye Y Gao J Liang J Yang Y Lv C Chen M . Association between preoperative lipid profiles and new-onset diabetes after transplantation in Chinese kidney transplant recipients: A retrospective cohort study. J Clin Lab Anal. (2021) 35:e23867. doi: 10.21203/rs.3.rs-93898/v1. PMID: 34101909 PMC8373348

[39] Cheng F Li Q Wang J Wang Z Zeng F Zhang Y . Analysis of risk factors and establishment of a risk prediction model for post-transplant diabetes mellitus after kidney transplantation. Saudi Pharm J. (2022) 30:1088–94. doi: 10.1016/j.jsps.2022.05.013. PMID: 36164572 PMC9508626

[40] Wang L Huang J Li Y Shi K Gao S Zhao W . Postoperative fasting plasma glucose and family history diabetes mellitus can predict post-transplantation diabetes mellitus in kidney transplant recipients. Endocrine. (2023) 81:58–66. doi: 10.1007/s12020-023-03374-y. PMID: 37148416

[41] Hasbal NB Copur S Peltek IB Mutlu A Atalay HO Kesgin YE . Pancreatic steatosis is an independent risk factor for post-transplant diabetes mellitus in kidney transplant patients. Clin Transplant. (2024) 38:e15204. doi: 10.1111/ctr.15204. PMID: 38041471

[42] Patel DD Modi KP Patel AK Chaudhary V . New onset of diabetes mellitus in Indian renal transplant recipient-a retrospective study. Int J Pharm Pharm Sci. (2015) 7:228–32.

[43] Bertram L Kasiske HAC Roel J . Explained and unexplained ischemic heart disease risk after renal transplantation. J Am Soc Nephrol. (2000) 11:1735–43. doi: 10.1681/asn.v1191735. PMID: 10966499

[44] Weng LC Chiang YJ Lin MH Hsieh CY Lin SC Wei TY . Association between use of FK506 and prevalence of post-transplantation diabetes mellitus in kidney transplant patients. Transplant Proc. (2014) 46:529–31. doi: 10.1016/j.transproceed.2013.11.141. PMID: 24656004

[45] Sood A Nadey DNH Hakim S . Consequences of recipient obesity on postoperative outcomes in a renal transplant a systematic review and meta-analysis. Exp Clin Transplant. (2016) 14:121–8. doi: 10.6002/ect.2015.0295 27015529

[46] Lafranca JA IJ JN Betjes MG Dor FJ . Body mass index and outcome in renal transplant recipients: a systematic review and meta-analysis. BMC Med. (2015) 13:111. doi: 10.1186/s12916-015-0340-5. PMID: 25963131 PMC4427990

[47] Ishikawa S Tasaki M Ikeda M Nakagawa Y Saito K Tomita Y . Pretransplant BMI should be <25 in Japanese kidney transplant recipients: A single-center experience. Transplant Proc. (2023) 55:72–9. doi: 10.1016/j.transproceed.2022.10.058. PMID: 36528408

[48] Cullen TJ McCarthy MP Lasarev MR Barry JM Stadler DD . Body mass index and the development of new-onset diabetes mellitus or the worsening of pre-existing diabetes mellitus in adult kidney transplant patients. J Renal Nutr. (2014) 24:116–22. doi: 10.1053/j.jrn.2013.11.002. PMID: 24411665

[49] Dong Y . Influencing factors of post-transplantation diabetes mellitus in kidney transplant recipients and establishment of a risk prediction model. Investigacion Clinica (Venezuela). (2023) 64:460–70. doi: 10.54817/ic.v64n4a03

[50] Heisel O Heisel R Balshaw R Keown P . New onset diabetes mellitus in patients receiving calcineurin inhibitors: a systematic review and meta-analysis. Am J Transplant. (2004) 4:583–95. doi: 10.1046/j.1600-6143.2003.00372.x. PMID: 15023151

[51] Wellen KE Hotamisligil GS . Inflammation, stress, and diabetes. J Clin Invest. (2005) 115:1111–9. doi: 10.1172/jci200525102. PMID: 15864338 PMC1087185

[52] Pradhan AD Manson JE Rifai N Buring JE Ridker PM . C-reactive protein, interleukin 6, and risk of developing type 2 diabetes mellitus. Jama. (2001) 286:327–34. doi: 10.1001/jama.286.3.327. PMID: 11466099

[53] McLaughlin T Reaven G Abbasi F Lamendola C Saad M Waters D . Is there a simple way to identify insulin-resistant individuals at increased risk of cardiovascular disease? Am J Cardiol. (2005) 96:399–404. doi: 10.1016/j.amjcard.2005.03.085. PMID: 16054467

[54] Du Q Li T Yi X Song S Kang J Jiang Y . Prevalence of new-onset diabetes mellitus after kidney transplantation: a systematic review and meta-analysis. Acta Diabetologica. (2024) 61(7):809–29. doi: 10.1007/s00592-024-02253-w. PMID: 38507083

[55] Gomes V Ferreira F Guerra J Bugalho MJ . New-onset diabetes after kidney transplantation: incidence and associated factors. World J Diabetes. (2018) 9:132–7. doi: 10.4239/wjd.v9.i7.132. PMID: 30079149 PMC6068739

[56] Ponticelli C Favi E Ferraresso M . New-onset diabetes after kidney transplantation. Med (Kaunas). (2021) 57:1–9. doi: 10.3390/medicina57030250. PMID: 33800138 PMC7998982

[57] Kanbay M Copur S Topçu AU Guldan M Ozbek L Gaipov A . An update review of post-transplant diabetes mellitus: concept, risk factors, clinical implications and management. Diabetes Obes Metab. (2024) 26:2531–45. doi: 10.1111/dom.15575. PMID: 38558257

[58] Sabharwal S Delgado-Borrego A Chung RT . Extrahepatic hepatitis C virus after transplantation: diabetes and renal dysfunction. Liver Transplant. (2008) 14:S51–7. doi: 10.1002/lt.21613. PMID: 18825714

[59] Bose SK . Hepatitis C virus infection and insulin resistance. World J Diabetes. (2014) 5:52-8. doi: 10.4239/wjd.v5.i1.52. PMID: 24567801 PMC3932427

[60] Masini M Campani D Boggi U Menicagli M Funel N Pollera M . Hepatitis C virus infection and human pancreatic beta-cell dysfunction. Diabetes Care. (2005) 28:940–1. doi: 10.2337/diacare.28.4.940. PMID: 15793203

[61] Culliford A Phagura N Sharif A . Autosomal dominant polycystic kidney disease is a risk factor for posttransplantation diabetes mellitus: an updated systematic review and meta-analysis. Transplant Direct. (2020) 6:1-8. doi: 10.1097/txd.0000000000000989. PMID: 32548247 PMC7213605

[62] Vincenti F Friman S Scheuermann E Rostaing L Jenssen T Campistol JM . Results of an international, randomized trial comparing glucose metabolism disorders and outcome with cyclosporine versus tacrolimus. Am J Transplant. (2007) 7:1506–14. doi: 10.1111/j.1600-6143.2007.01749.x. PMID: 17359512

[63] Chakkera HA Kudva Y Kaplan B . Calcineurin inhibitors: pharmacologic mechanisms impacting both insulin resistance and insulin secretion leading to glucose dysregulation and diabetes mellitus. Clin Pharmacol Ther. (2017) 101:114–20. doi: 10.1002/cpt.546. PMID: 27804122

[64] Cheng F Li Q Wang J Hu M Zeng F Wang Z . Genetic polymorphisms affecting tacrolimus metabolism and the relationship to post-transplant outcomes in kidney transplant recipients. Pharmgenomics Pers Med. (2021) 14:1463–74. doi: 10.2147/pgpm.s337947. PMID: 34824543 PMC8610755

[65] Jenssen T Hartmann A . Post-transplant diabetes mellitus in patients with solid organ transplants. Nat Rev Endocrinol. (2019) 15:172–88. doi: 10.1038/s41574-018-0137-7. PMID: 30622369

[66] Li JX Cummins CL . Fresh insights into glucocorticoid-induced diabetes mellitus and new therapeutic directions. Nat Rev Endocrinol. (2022) 18:540–57. doi: 10.1038/s41574-022-00683-6. PMID: 35585199 PMC9116713

