# Supplementary material for: Incidence and risk factors of post-transplant diabetes mellitus after kidney transplantation: a systematic review and meta-analysis
Source: Front Endocrinol (Lausanne). 2026 May 25;17:1838424. doi: 10.3389/fendo.2026.1838424 (PMC13243380; doi:10.3389/fendo.2026.1838424)
Supplement: Supplementary file 1 [file Table1.docx]

**Table S1: Search strategy**

**Embase**

ID Search

#1 'kidney transplantation'/exp OR 'renal transplantation*':ab,ti OR 'kidney graft*':ab,ti OR 'kidney transplant*':ab,ti

#2 'diabetes mellitus'/exp OR diabet*:ab,ti

#3 'influence factor*':ab,ti OR 'risk factor':ab,ti OR 'predictive factor*':ab,ti OR incidence:ab,ti OR incidence*:ab,ti

#4 'cohort analysis'/exp OR 'cohort studie*':ab,ti OR 'concurrent stud*':ab,ti OR 'incidence stud*':ab,ti OR 'prospective study'/exp OR 'prospective studie*':ab,ti OR 'retrospective study'/exp OR 'retrospective studie*':ab,ti OR 'ex post facto design':ab,ti

#5 post:ab,ti OR newonset:ab,ti

#6 #1 AND #2 AND #3 AND #4 AND #5

**Pubmed**

**Search Query**

**#1** (((Kidney Transplantation[MeSH Terms]) OR (Renal Transplantation*[Title/Abstract])) OR (kidney graft*[Title/Abstract])) OR (kidney transplant*[Title/Abstract])

**#2** (diabetes mellitus[MeSH Terms]) OR (Diabet*[Title/Abstract])

**#3** ((influence factor*[Title/Abstract]) OR (Risk factor[Title/Abstract])) OR (Predictive factor*[Title/Abstract])

**#4** (incidence[MeSH Terms]) OR (Incidence*[Title/Abstract])

**#5** ((((((((cohort studies[MeSH Terms]) OR (Cohort Studie*[Title/Abstract])) OR (Concurrent Stud*[Title/Abstract])) OR (Incidence Stud*[Title/Abstract])) OR (prospective studies[MeSH Terms])) OR (Prospective Studie*[Title/Abstract])) OR (Retrospective Studies[MeSH Terms])) OR (Retrospective Studie*[Title/Abstract])) OR (ex post facto design[Title/Abstract])

**#6** (Post[Title/Abstract]) OR (Newonset[Title/Abstract])

**#7** #1 and #2 and #3 or #4 and #5 and #6

**Cochrane**

ID Search

#1 MeSH descriptor: [Kidney Transplantation] explode all tree

#2 (Renal Transplantation*):ti,ab,kw OR (kidney graft*):ti,ab,kw OR (kidney transplant*):ti,ab,kw

#3 MeSH descriptor: [Diabetes Mellitus] explode all trees

#4 (Diabet*):ti,ab,kw

#5 (influence factor*):ti,ab,kw OR (Risk factor):ti,ab,kw OR (Predictive factor*):ti,ab,kw

#6 MeSH descriptor: [Incidence] explode all trees

#7 (Incidence*):ti,ab,kw

#8 MeSH descriptor: [Cohort Studies] explode all trees

#9 (Cohort Studie*):ti,ab,kw OR (Concurrent Stud*):ti,ab,kw OR (Incidence Stud*):ti,ab,kw

#10 MeSH descriptor: [Prospective Studies] explode all trees

#11 MeSH descriptor: [Retrospective Studies] explode all trees

#12 (Prospective Studie*):ti,ab,kw OR (Retrospective Studie*):ti,ab,kw OR (ex post facto design):ti,ab,kw

#13 #1 or #2

#14 #3 or #4

#15 #5 or #6 or #7

#16 #8 or #9 or #10 or #11 or #12

#17 #13 and #14 and #14 and #15

**Web of Science**

1: Renal Transplantation* (Topic) or kidney graft* (Topic) or kidney transplant* (Topic)

2: diabetes mellitus (Topic) or Diabet* (Topic)

3: influence factor* (Topic) or Risk factor (Topic) or Predictive factor* (Topic) or Incidence* (Topic)

4: cohort analysis (Topic) or Cohort Studie* (Topic) or Concurrent Stud* (Topic) or Incidence Stud* (Topic) or prospective study (Topic) or Prospective Studie* (Topic) or retrospective study (Topic) or Retrospective Studie* (Topic) or ex post facto design (Topic)

5: Post (Topic) or Newonset (Topic)

6: #1 AND #2 AND #3 AND #4 AND #5

**Table S2** Baseline characteristics of the included studies-PICOS

|  | Country | Study design | **Definition of NODAT** | Sample size,n  (T/C) | Age,years  Mean±SD  (T) | Time to diagnosis of new onset diabetes | Incidence rate | Risk faction |
| --- | --- | --- | --- | --- | --- | --- | --- | --- |
| Augusto2014 | France | cohort study | ADA | 28/154 | 47.3 | 1 year | age、sex、race、BMI、DV、RRT、donor age、AR、CIT、ON、diuretic、IR、Mg、Glu、TC、TG、HbA1c | Glu、TG、Mg |
| Ivarsson2014 | Sweden | cohort study | resident physician | 32/222 | 53.9 | ≥1 year | age、sex、BMI、ON、RRT、DV、DT、IR、PTH | age、sex、PTH |
| Dedinská2015 | Europe | cohort study | ADA | 64/167 | 52 | 12 months | age、 weight、BMI、waist circumference、TG、TC、IR | age、waist circumference、IR |
| Aleid2016 | Saudi Arabia | cohort study | ICG 2003 | 136/500 | 45 | ≥1 year | DT、sex、smoking、DGF、ON、outcomes、age、FHOD、Glu、HCV、AR | age、FHOD、HCV、FPG、IFG |
| Alshamsi2016 | Saudi Arabia | cohort study | ADA | 43/279 | 43.6 | 3 years | age、Height、weight、BMI、FPG、Cr | BMI、FHOD、age、FPG、sex、DT |
| Cheng2016 | China | cohort study | ADA | 20/197 | 48.1 | 3 years | age、sex、BMI、smoking、alcoholism、FHOD、HBV、Hypertension、Glu、Liver function（ALT、AST、ALP、GGT、T-BiL、D-BiL)、TC、TG、HDL-C、LDL-C、IR 、AR、CMV | age、FHOD、FPG、ALT、TC、HDL-C、ALP |
| Dedinská2016 | Slovak Republic | cohort study | ADA | 64/167 | 50.5 | 12 months | age、sex、HLA、ADPKD、DT、CMV、IR | age、IR |
| Dedinská2016 | Slovak Republic | cohort study | ADA | 52/133 | 51.5 | 12 months | age、sex、Prediabetes、ADPKD、FHOD、Hypertension、HLA、waist circumference、BMI、IR、C-peptide、IRI、TG、TC、HbA1c、Mg、HOMA-IR | age、FHOD、waist circumference、BMI、C-peptide、IRI、TG、TC |
| PATEL2016 | India | cohort study | author | 23/77 | 37.91 | 12 months | age、sex、FHOD、BMI、HCV、TC、TG、CMV、AR、IR | age、FHOD、BMI、HCV、TC、TG |
| Sinangil2016 | Istanbul | cohort study | ADA | 70/419 | 51.7 | ≥12 months | age、sex、BMI、FUT、FPG、AR、IR | age、BMI、Mg |
| Agrawal2018 | Nepal | cohort study | ADA | 22/97 | 33.5 | 62.7±43.4 days | age、sex、Blood group、BMI、ON、DV、Hemoglobin、Glu、donor age、DT、HLA | age、sex、Blood group、BMI、ON、DT、HLA、DV、IR |
| Kumar2018 | India | cohort study | ADA | 24/100 | 45.2 | 1 year | age、BMI、FHOD、HBV、HCV、ADPKD、Mg、TC、TG、HbA1c、Blood group、HOMA-IR、HOMA-S、HOMA‑beta-cell function、C-peptide | age、BMI、FHOD、HBV、HCV、ADPKD、Mg、TC;、TG、HbA1c、Blood group、HOMA-IR、HOMA-S、HOMA‑beta-cell function、C-peptide |
| Biró2019 | Hungary | cohort study | ADA | 33/223 | 54.2 | 2 years | sex、age、BMI、DT、RRT、DGF、CMV、Hypertension、ADPKD、TC、LDL-C、HDL-C、TG、FPG、Glu、AFPG、IR | DT、RRT、DGF、CMV、Hypertension、ADPKD、TC、LDL-C、HDL-C、TG、FPG、Glu、AFPG、IR |
| Cai2019 | China | cohort study | ADA | 166/633 | 54 | 1 year | age、sex、smoking、FHOD、RRT、HBV、HCV、Time to PTDM、Antihypertensive medication、Statin medication、Metabolic syndrome 、FPG、HDL-C、TG、BMI、SBP、DBP | BMI |
| Lai2019 | China | cohort study | ADA | 36/290 | 45.2 | 1 year | age、sex、BMI、RRT、ON、DT、IR、HbA1c、ALB、TG、TC、CPI | age、BMI、ON、DT、HbA1c、TC、CPI |
| Paek2019 | Korea | cohort study | ADA | 85/723 | 43.8 | 1 year | age、sex、smoking、Hypertension、Dyslipidemia、HCV；donor age、DT、DM、donor Hypertension、HLA、Presence of DSA、BMI、Waist-hip ratio、HbA1c、Glu、Ferritin、Transferrin saturation、IR | AR、BMI、HLA、Transferrin saturation、age、Waist-hip ratio、HbA1c |
| Tomkins2019 | Ireland | cohort study | ADA | 38/347 | 57 | 10 years | sex、age、DT、 weight、HbA1c、smoking | age、sex、 weight，donor age、donor sex |
| Ajabnoor2020 | Saudi Arabia | cohort study | ADA | 53/235 | 44 | 10 months | age、sex、BMI、FHOD、HCV、RRT、DT、Number of transplants、Prediabetes、LDL-C、TG | age、BMI、IR |
| van der Burgh2020 | Netherlands | cohort study | ADA | 29/167 | 60 | 1 year | age、sex、BMI、SBP、DBP、ON、eGFR、Glu、Mg、IR、Urine Mg/Cr | age、BMI、IR、Mg |
| Malik2021 | USA | cohort study | ADA | 186/632 | 55.24 | 5 years | age、race、sex、weight、BMI、Previous kidney transplant、DV、ON、Preemptive transplant、transfusions、HCV、HLA、PPRA、Kidney pumped、Kidney biopsied、CIT | age、BMI、race、HLA |
| Ye2021 | China | cohort study | ADA | 104/449 | 42.26 | 28.03 months | sex、age、BMI、TC、TG、HDL-C、LDL-C、FPG、Cr、FUT、FHOD、ADPKD、lipid-lowering drugs、HCV、CMV、DT、AR、IL-2Ra、IR | sex、age、BMI、TC、TG、HDL-C、LDL-C、FPG、Hypertension、FUT、FHOD、ADPKD、lipid-lowering drugs、HCV、CMV、DT、AR、IL-2Ra、IR |
| Cheng2022 | China | cohort study | ADA | 55/495 | 46 | ≥1 months | sex、age、BMI、FUT、Hypertension、Anemia、HBV、Coronary heart disease、Arthrolithiasis、IR | age、BMI、IR 、hyperglycaemia、DGF、AR |
| Wang2023 | China | cohort study | WHO | 38/536 | 41.5 | 12 months | age、sex、Height、weight、BMI、smoking、FHOD、CVD、HCV、ASA grade III、ON、RRT、BUN、Cr、UA、RPG、TC、IR | FHOD、FPG |
| Hasbal2024 | Türkiye | cohort study | ADA | 69/373 | 48.2 | From 45 days after transplantation | age、sex、BMI、smoking、FHOD、Hypertension、Heart failure、ALT、AST、ALP、Total bilirubin、ALB、Liver fibrosis-4 score、Nonalcoholic fatty liver disease fibrosis score、Hemoglobin、Platelets、Cr、BUN、eGFR、Proteinuria、Glu、UA、Mg、TSH、TC、TG、HDL-C、LDL-C、HCV、HBV、CMV、donor CMV、hepatic steatosis、pancreatic steatosis、graft rejection | age、Pancreatic steatosis、TG、Glu |
| Jiang2024 | China | cohort study | ADA | 37/409 | 44.5 | 68 days | sex、age、BMI、HBV、HCV、Mg、Proteinuria、SBP、DBP、Glu、TC、TG、HDL-C、LDL-C、Urea 、UA、Cr | age、BMI、IR、antibiotic use |
| Ünlütürk2024 | Türkiye | cohort study | ADA | 101/703 | 39 | 12 months | age、sex、BMI、Glu、HbA1c、antihyperglycemic drug、LDL-C、Proteinuria、RRT、DV、DT、IR 、HBV、HCV、CMV、rejection、cardiovascular events | BMI、FPG、HCV |

BMI, body mass index; DV, Dialysis vintage; RRT, renal replacement therapy; AR, Acute rejection; CIT, Cold ischemia time; ON, Original nephropathy; IR, Immunosuppressive regimen; Mg, magnesium; Glu, glucose; TC, total cholesterol; TG, triglycerides; HbA1c, glycated hemoglobin; DT, Donor type; PTH, parathyroid hormone; DGF, Delayed graft function；FHOD, Family history of diabetes; HCV, hepatitis C virus; FPG, fasting plasma glucose; IFG, Impaired fasting glucose; Cr, creatinine; HBV, hepatitis B virus; ALT, alanine aminotransferase; AST, aspartate aminotransferase; ALP, alkaline phosphatase; GGT, gamma-glutamyltransferase; T-BiL, total bilirubin; D-BiL, direct bilirubin; HDL-C, high-density lipoprotein-cholesterol; LDL-C, low-density lipoprotein cholesterol; CMV, cytomegalovirus; HLA, Human Leukocyte Antigen; ADPKD, autosomic dominant polycystic kidney disease; IRI, immunoreactive insulin;

HOMA-IR, Homeostatic Model Assessment of Insulin Resistance; FUT, Follow-up time; HOMA-S, insulin sensitivity; AFPG, averaged fasting plasma glucose during the first post-transplant week; PTDM, Post-transplantation diabetes mellitus; SBP, systolic blood pressure; DBP, diastolic blood pressure; DM, Diabetes mellitus; AC, Abdominal circumference; DSA, donor specific antibody; BFA, Body fat area; SFA, Subcutaneous fat area; VFA, Visceral fat area; eGFR ,estimated glomerular filtration rate; PPRA, Peak panel reactive antibody; IL-2Ra, interleukin-2 receptor antagonists; CVD，cardiovascular disease；ASA, ASA American Society of Anesthesiologists physical status classification system; BUN, blood urea nitrogen; UA, Uric acid; RPG, random plasma glucose; ALB, albumin; TSH, thyroid-stimulating hormone;

**Table S3** NOS quality scores of the included cohort Studies

| **Cohort Studies** | **Selection of the research population** | | | | **Comparability** | **Outcome** | | | **Score** |
| --- | --- | --- | --- | --- | --- | --- | --- | --- | --- |
|  | **Representativeness of the Exposed Cohort** | **Selection of the Non-Exposed Cohort** | **Ascertainment of Exposure** | **Demonstration That Outcome of Interest Was Not Present at Start of Study** |  | **Assessment of Outcome** | **Follow-Up Duration** | **Adequacy of Follow Up** |  |
| Augusto2014 | 1 | 1 | 1 | 1 | 2 | 1 | 1 | 1 | 9 |
| Ivarsson2014 | 1 | 1 | 1 | 1 | 2 | 0 | 1 | 1 | 8 |
| Dedinská2015 | 1 | 1 | 1 | 1 | 2 | 1 | 1 | 1 | 9 |
| Aleid2016 | 1 | 1 | 1 | 1 | 2 | 1 | 1 | 1 | 9 |
| Alshamsi2016 | 1 | 1 | 1 | 1 | 2 | 1 | 1 | 0 | 8 |
| Cheng2016 | 1 | 1 | 1 | 1 | 2 | 1 | 1 | 1 | 9 |
| Dedinská2016 | 1 | 1 | 1 | 1 | 1 | 1 | 1 | 1 | 8 |
| Dedinskáa2016 | 1 | 1 | 1 | 1 | 2 | 1 | 1 | 1 | 9 |
| PATEL2016 | 1 | 1 | 1 | 1 | 2 | 0 | 1 | 1 | 8 |
| Sinangil2016 | 1 | 1 | 1 | 1 | 2 | 1 | 1 | 1 | 9 |
| Agrawal2018 | 1 | 1 | 1 | 1 | 2 | 1 | 0 | 0 | 7 |
| Kumar2018 | 1 | 1 | 1 | 1 | 1 | 1 | 1 | 1 | 8 |
| Biró2019 | 1 | 1 | 1 | 1 | 2 | 1 | 1 | 0 | 8 |
| Cai2019 | 1 | 1 | 1 | 1 | 2 | 1 | 1 | 1 | 9 |
| Lai2019 | 1 | 1 | 1 | 1 | 2 | 1 | 1 | 0 | 8 |
| Paek2019 | 1 | 1 | 1 | 1 | 2 | 1 | 1 | 1 | 9 |
| Tomkins2019 | 1 | 1 | 1 | 1 | 2 | 1 | 1 | 1 | 9 |
| Ajabnoor2020 | 1 | 1 | 1 | 1 | 1 | 1 | 1 | 1 | 8 |
| van der Burgh2020 | 1 | 1 | 1 | 1 | 2 | 1 | 1 | 1 | 9 |
| Malik2021 | 1 | 1 | 1 | 1 | 2 | 1 | 1 | 1 | 9 |
| Ye2021 | 1 | 1 | 1 | 1 | 2 | 1 | 1 | 1 | 9 |
| Cheng2022 | 1 | 1 | 1 | 1 | 2 | 1 | 1 | 1 | 9 |
| Wang2023 | 1 | 1 | 1 | 1 | 2 | 0 | 1 | 1 | 9 |
| Hasbal2024 | 1 | 1 | 1 | 1 | 2 | 1 | 1 | 1 | 9 |
| Jiang2024 | 1 | 1 | 1 | 1 | 1 | 1 | 1 | 1 | 8 |
| Ünlütürk2024 | 1 | 1 | 1 | 0 | 2 | 1 | 0 | 1 | 7 |
